# Influence of composite flour constituents and extrusion temperature in the production of analog rice

**DOI:** 10.1002/fsn3.2411

**Published:** 2021-07-07

**Authors:** Siswo Sumardiono, Budiyono, Heny Kusumayanti, Novian Indra Agung Prakoso, Fawzia Puti Paundrianagari, Heri Cahyono

**Affiliations:** ^1^ Department of Chemical Engineering Faculty of Engineering Universitas Diponegoro Semarang Indonesia; ^2^ Department of Industrial Chemical Engineering Vocational School Universitas Diponegoro Semarang Indonesia

**Keywords:** analog rice, calcium, composite flour, extrusion, snakehead fish

## Abstract

This study was conducted to determine the effect of composite flour (CF) constituents and different extrusion temperatures on the production of analog rice and community perceptions about the produced rice. CF was produced by mixing modified cassava flour (Mocaf) and snakehead fish flour (SF) in the following ratios: 100:0; 97:3; 94:6; 91:9; and 88:12. Analog rice was then extruded from CF at the following temperatures: 50, 70, and 90°C. The analog rice was tested for physical properties (bulk density and cooking time), chemical properties (nutrient content), and organoleptic properties. The results showed that CF and extrusion temperature affect the nutrient content of analog rice. The best analog rice formulation constituted of CF with Mocaf:SF ratio of 91:9 and extrusion temperature of 70°C, and contained 14.34% water, 0.85% ash, 71.83% carbohydrate, 11.24% protein, 1.12%, calcium 1,113 ppm, and 2.43% dietary fiber. This study included 42 respondents aged 20–23 years, including 20 males and 22 females. All the respondents showed acceptance for the analog rice, suggesting that it could substitute ordinary rice as a staple food.

## Graphical abstract

This study was conducted to determine the effect of composite flour constituents and different extrusion temperatures on the production of analog rice and community perceptions on the produced rice. Composite flour (CF) was produced by mixing modified cassava flour (Mocaf) and snakehead fish flour (SF) in the following ratios: 100:0; 97:3; 94 6; 91:9; and 88:12. Analog rice was then extruded from the CF at the following temperatures: 50, 70, and 90°C. The best analog rice formulation constituted of composite flour with Mocaf–SF ratio of 91:9 and extrusion temperature of 70°C

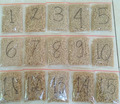

## 1 INTRODUCTION

Rice consumption is increasing every year in line with the increasing world population. The nutritional composition of white rice is 40%–80% calories, 78.9% carbohydrate, 6.8% protein, and 0.8% iron (Dalbhagat et al., 2019). As the world's population is increasing, so is the proportion of elderly people. One of the diseases that are familiar to the elderly is osteoporosis, caused by bone metabolism disorder. Various conditions can disrupt bone metabolism: reduced estrogen, decreased calcium, and decreased mechanical stimulation. Calcium intake can be increased by adding these minerals to commonly consumed foods such as rice by fortification of food. Food fortification is essential to add one or more nutrients to food (Putri & Sumardiono, 2020; Santosa et al., 2017). Foodstuffs used as a fortification matrix can produce either ready‐to‐eat food or raw materials that require processing (Wardhani et al., 2018). Fortification programs serve as a low‐cost solution that addresses nutrient deficiencies (Santosa et al., 2017).

A very high dependence of the Indonesian people on rice and a need for high calcium food for the elderly are prevailing problems. Therefore, new solutions are needed to overcome these problems, one of which is producing analog rice. Analog rice is an alternative food to substitute rice shaped like a grain of rice produced from corn, sago, cassava, sorghum, composite flour, and other materials. It has characteristics like rice, physical properties of grain, and good mixture and texture. Analog rice has nutrients that can be adjusted to be higher than real rice (Sumardiono et al., 2018). Previous studies have been undertaken to attempt to make analog rice from purple sweet potato (Handayani et al., 2017); cassava, corn, and taro (Pudjihastuti et al., 2019); modified cassava and corn (Sumardiono et al., 2018); cassava, green bean, and hanjeli (Sumardiono et al., 2014); and cassava, avocado seeds, and tofu waste (Putri & Sumardiono, 2020).

Snakehead fish is one of the high calcium foods that can meet the nutritional needs of the elderly. Every 100 g of snakehead fish contains at least 29 mg of calcium, 124 mg of phosphorus, and 0.64 mg of iron (Asfar et al., 2014). Snakehead fish is relatively easy to find, but not all households can process snakehead fish well to be easily consumed by the elderly. Therefore, an alternative product is needed that contains enough nutrients as a substitute for the usually processed snakehead fish. One effort to create the substitution product is by making the staple food of fortified analog rice.

The combination of cassava, corn, and snakehead fish will create analog rice with high calcium content, where snakehead fish is the source of calcium.

This research aimed to study the process of analog rice production by varying the constituents of composite flour (CF; cassava, corn, and snakehead fish) and different extrusion temperatures. The study also aimed at determining respondents' perception of the best analog rice and its nutritional content.

## 2 MATERIALS AND METHODS

### 2.1 Materials

The materials used in this study were modified cassava flour/Mocaf from Omah Tani, Gunung Pati, Semarang, Central Java; snakehead fish meat from a fish market in Semarang, Indonesia; corn starch (CS); and additional ingredients (flour, glycerol monostearate, water, and cooking oil). Other chemicals used in this work were of analytical grade and used directly without pretreatment.

### 2.2 Making of snakehead fish flour (SF)

SF was made using a modified procedure by Widodo et al. (2015). Cleaned snakehead fish meat was boiled for 15 min with water at the ratio of 1:1, drained, and weighed. The fish flesh was further separated from the bone and skin. The antioxidant butylated hydroxytoluene was added up to 0.02% of the weight of the fish meat to the broth and mixed well. The fish meat was pulverized, mixed with fish broth, and weighed. The fish pulp was dried at 50°C for 9 hr. Dried fish pulp was mashed with the food processor; subsequently, the solid components were sifted using an 80‐mesh sieve.

### 2.3 Making of CF

CF was prepared by mixing Mocaf and SF flour in the following ratios(%w) (100:0; 97:3; 94:6; 91:9; 88:12) with a basis weight of 280 g; then, 120 g CS flour was added to each mixed flour.

### 2.4 Extrusion of analog rice

CF dough was obtained by mixing each sample of CF flour with 400 ml water and 4 g glycerol monostearate. The dough was wrapped and compacted in a cloth, then steamed for 30 min with a temperature of approximately 80°C. The dough that had been preconditioned was placed in the extruder. The extruder used in this study is a single screw extruder equipped with a heating controller assembled from CV Teguh Jaya Teknik Ungaran, Semarang, Indonesia. The extruder operating temperatures were set at (50; 70; and 90°C) for constituents of composite flour variable and (50, 60, 70, 80, and 90°C) for extrusion temperature variable. After extrusion, the wet analog rice grains produced were dried at room temperature for 24 hr (Faleh et al., 2013).

### 2.5 Analytical methods

Proximate analysis was done on the analog rice to determine carbohydrate content, ash content, and water content, Fehling solution was used to obtain the glucose concentration, fat content was determined by the extraction method, and Kjeldahl analysis was used to determine protein and calcium concentration (AOAC, 2005).

### 2.6 Sensory analysis

Hedonic test was performed to determine the preference of analog rice by respondents. The hedonic scale consisted of the following five parameters: very much disliked (1), did not like (2), neutral (3), liked (4), and very much liked (5). Forty‐two people were selected for the hedonic test (average age range: 20–23 years, 20 males and 22 females) at the Department of Chemical Engineering, Universitas Diponegoro. The parameters assessed include aroma, color, taste, and texture.

### 2.7 Statistical analysis

Data processing for proximate analyses, calcium analysis, and hedonic rating was performed using one‐way analysis of variance, and the Duncan post hoc test was used to separate means.

## 3 RESULTS AND DISCUSSION

### 3.1 Proximate analysis of raw material and analog rice

**FIGURE 1:**
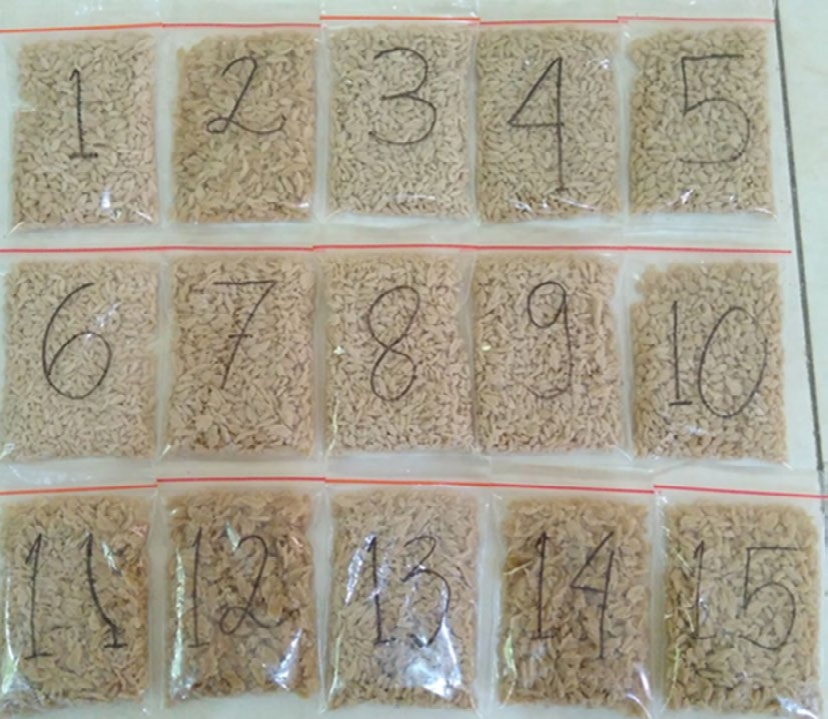
Fortified analog rice

The result of the analysis is represented by Tables 1 and 2. The appearance of analog rice variations of sample 1–15 is shown in Figure 1. Testing the nutrition of raw materials in this research has the same results as the nutritional content conducted by several previous studies (Onyango et al., 2020; Nadimin & Lestari, 2019; Suarni et al., 2013). The proximate test of modified cassava flour obtained the same results as previous research conducted, where the starch content was around 80% (Onyango et al., 2020; Sumardiono et al., 2017; Sumardiono et al., 2017). Nadimin & Lestari, 2019 researching the content of snakehead fish flour confirmed that the relatively high calcium content and proximate content were relatively not much different from the raw material for snakehead fish flour in this study. The proximate of corn starch in this research has the same results with the research conducted by Rahmawati and Yaniansah (2021) testing the proximate of various maize varieties in Indonesia.

**TABLE 1:** Proximate analysis of analog rice raw materials

Sample	Calcium (ppm)	Carbohydrate (wt.%)	Protein (wt.%)	Fat (wt.%)	Dietary fiber (wt.%)	Moisture (wt.%)	Ash (wt.%)
Modified cassava flour	33 ± 0.09	83.22 ± 0.6	3.73 ± 0.3	0.14 ± 0.005	6.47 ± 0.1	5.10 ± 0.06	1.80 ± 0.02
Snakehead fish flour	1,361 ± 0.06	38.05 ± 0.4	54.56 ± 1.1	0.06 ± 0.001	6.31 ± 0.09	0.68 ± 0.004	0.84 ± 0.003
Corn starch	20 ± 0.04	80.37 ± 0.3	9.11 ± 0.1	1.54 ± 0.003	5.42 ± 0.2	2.99 ± 0.09	0.58 ± 0.001

**TABLE 2:** Proximate analysis of analog rice (wt.%/100 g)

Sample	Ratio		Temperature of extrusion (^o^C)	Calcium (ppm)	Carbohydrate (wt.%)	Protein (wt.%)	Fat (wt.%)	Dietary fiber (wt.%)	Moisture (wt.%)	Ash (wt.%)
	Modified cassava flour (%)	Snakehead fish flour (SF) (%)								
Modified cassava flour	100	0	0	33 ± 0.09^j^	71.82 ± 0.2^c^	7.95 ± 0.02^f^	2.18 ± 0.003^a^	7.05 ± 0.06^a^	11.00 ± 0.04^g^	1.80 ± 0.02^a^
1	100	0	50	676.50 ± 7.2^g^	70.73 ± 0.4^cd^	7.21 ± 0.01^g^	0.64 ± 0.002^f^	4.22± 0.02^b^	16.50 ± 0.02^a^	0.70 ± 0.001^e^
2	97	3		717.50 ± 10.4^f^	68.76 ± 0.5^e^	8.50 ± 0.02^ef^	0.72 ± 0.001^e^	4.08 ± 0.04^c^	16.47 ± 0.05^a^	1.44 ± 0.003^b^
3	94	6		832.50 ± 8.2^e^	70.10 ± 0.2^d^	8.78 ± 0.03^e^	0.54 ± 0.002^g^	3.98 ± 0.06^d^	16.49 ± 0.04^a^	0.10 ± 0.001^g^
4	91	9		908.00 ± 6.7^bc^	70.31 ± 0.7^d^	7.97 ± 0.01^f^	0.62 ± 0.003^f^	3.80 ± 0.03^de^	16.37 ± 0.02^ab^	0.80 ± 0.002^d^
5	88	12		887.00 ± 3.2^c^	68.01 ± 0.7^e^	11.64 ± 0.04^a^	1.12 ± 0.001^c^	2.23 ± 0.009^h^	16.36 ± 0.06^ab^	0.50 ± 0.002^f^
6	100	0	70	713.00 ± 8.1^g^	72.35 ± 0. 2^bc^	7.62 ± 0.02^f^	0.92 ± 0.001^d^	3.92 ± 0.02^d^	14.34 ± 0.04^c^	0.85 ± 0.004^d^
7	97	3		662.50 ± 4.7^i^	70.79 ± 0.4^cd^	11.47 ± 0.01^a^	0.92 ± 0.001^d^	2.48 ± 0.03^g^	13.29 ± 0.06^e^	1.05 ± 0.006^c^
8	94	6		673.00 ± 11.5^h^	69.74 ± 0.6^de^	8.68 ± 0.01^e^	0.84 ± 0.001^de^	3.46 ± 0.02^ef^	15.41 ± 0.03^b^	0.85 ± 0.001^d^
9	91	9		1,113.00 ± 10.4^a^	71.83 ± 0.7^c^	11.24 ± 0.03^b^	1.12 ± 0.002^c^	2.43 ± 0.02^g^	13.53 ± 0.07^d^	0.85 ± 0.003^d^
10	88	12		976.50 ± 3.4^b^	71.25 ± 0.3^c^	9.04 ± 0.04^d^	0.66 ± 0.003^f^	3.36 ± 0.01^ef^	14.34 ± 0.06^c^	1.35 ± 0.002^b^
11	100	0	90	673.50 ± 5.6^g^	73.57 ± 0.5^b^	9.03 ± 0.02^d^	1.00 ± 0.002^cd^	3.68 ± 0.02^e^	11.93 ± 0.02^f^	0.80 ± 0.002^d^
12	97	3		881.50 ± 4.7^cd^	74.68 ± 0.2^a^	7.64± 0.01^f^	0.60 ± 0.004^f^	4.41 ± 0.04^b^	11.88 ± 0.04^f^	0.75 ± 0.003^e^
13	94	6		732.00 ± 1.1^f^	73.18 ± 0.09^b^	9.08 ± 0.01^d^	0.82 ± 0.003^de^	4.05 ± 0.03^c^	11.90± 0.05^f^	0.95 ± 0.001^cd^
14	91	9		892.50 ± 7.5^c^	73.21± 0.4^b^	9.37 ± 0.04^c^	0.90 ± 0.001^d^	3.69 ± 0.05^e^	11.88 ± 0.04^f^	0.90 ± 0.001^cd^
15	88	12		871.50 ± 5.5^d^	72.51 ± 0.1^bc^	10.01 ± 0.03^bc^	1.28 ± 0.002^b^	3.17 ± 0.02^f^	11.87 ± 0.06^f^	1.10 ± 0.002^c^

Note

Values are mean ± standard deviation. The values followed by different letters (a, b, c, d, e, f, g, h, i, j) in the same column are statistically different; *P* < .05, *n* = 3.

### 3.2 CF composition

**FIGURE 2:**
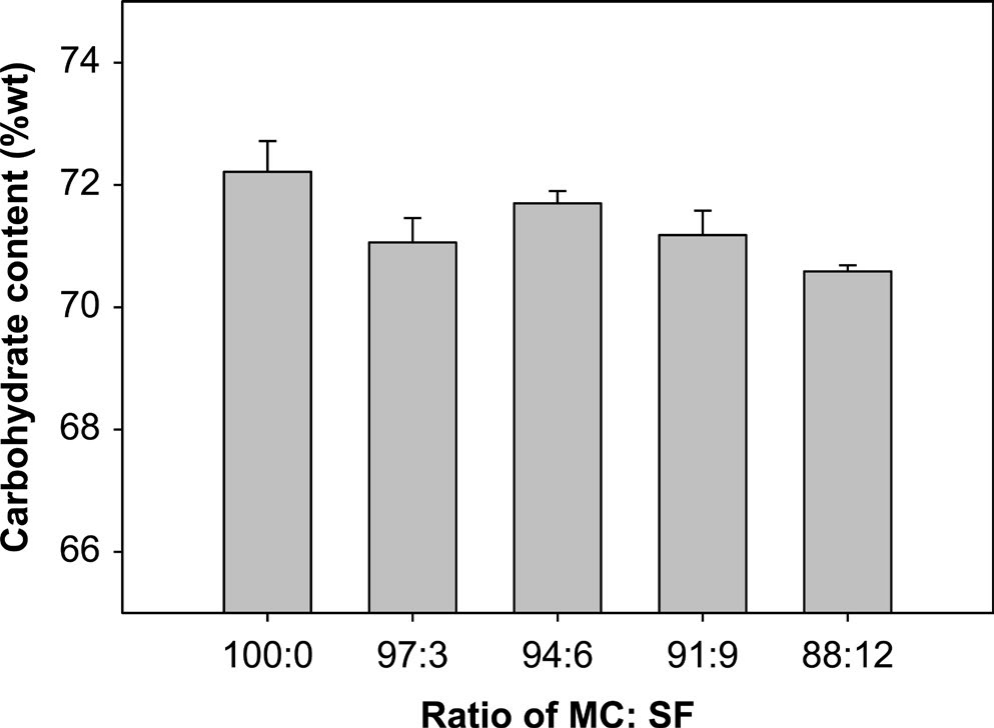
Influence of different composite flour formulations on carbohydrate content (at 70°C)

Table 3 shows the proximate analysis data for each CF ratio. They were statistical differences in carbohydrate content among the CF formulations. The CF with only Mocaf had the highest carbohydrate content (74.68%), while the CF formulation with a Mocaf:SF ratio of 88:12 had the least (68.01%), as shown in Figure 2.

**TABLE 3:** Nutritional content of different formulations of composite flour (at 70°C)

Parameter	Ratio of Modified Cassava Flour:Snakehead Fish Flour				
	100:0	97:3	94:6	91:9	88:12
Protein (% wt)	7.95 ± 0.2	9.20 ± 0.02	8.85 ± 0.03	9.53 ± 0.01	10.23 ± 0.02
Carbohydrate (% wt)	72.22 ± 0.01	71.06 ± 0.7	71.70 ± 0.6	71.18 ± 0.4	70.59 ± 0.3
Fat (% wt)	0.85 ± 0.002	0.75 ± 0.003	0.73 ± 0.003	0.88 ± 0.006	1.02 ± 0.007
Moisture (% wt)	14.25 ± 0.04	14.25 ± 0.02	14.25 ± 0.02	14.25 ± 0.05	14.25 ± 0.05
Ash (% wt)	0.78 ± 0.002	1.08 ± 0.003	0.63 ± 0.003	0.85 ± 0.005	0.98 ± 0.003
Calcium (ppm)	713.00 ± 8.1	662.50 ± 4.7	673.00 ± 11.5	1,113.00 ± 10.4	976.50 ± 3.4
Dietary Fiber (% wt)	3.94 ± 0.03	3.659 ± 0.03	3.83 ± 0.06	3.30 ± 0.02	2.92 ± 0.01

**FIGURE 3:**
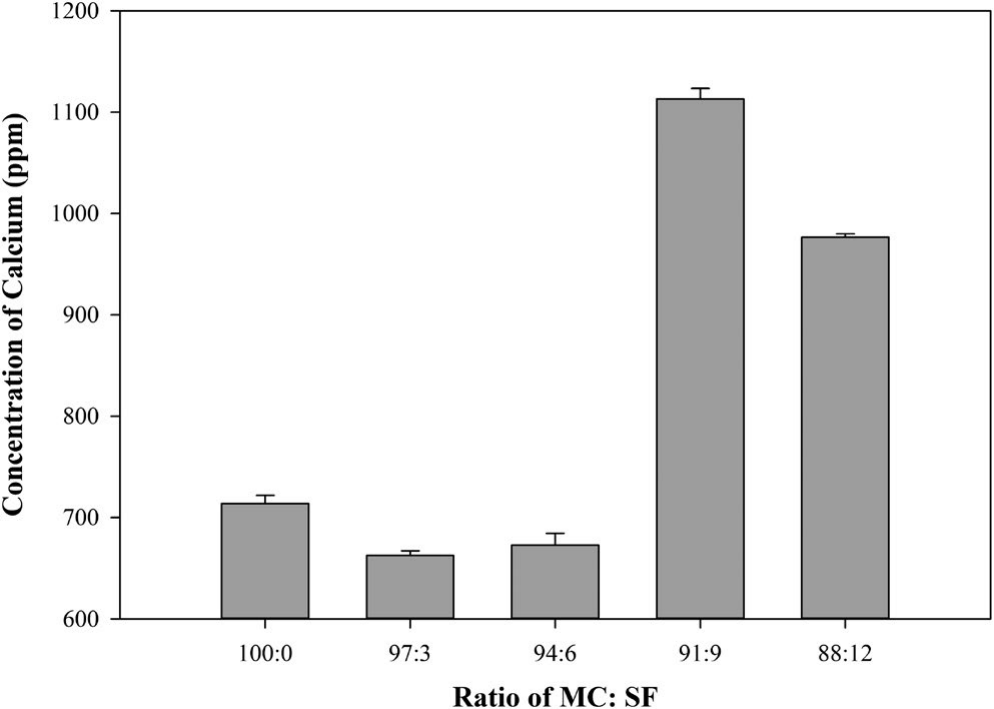
Influence of different composite flour formulations on calcium concentration (at 70°C)

There were statistical differences in calcium content among the CF formulations; the CF formulation with Mocaf:SF ratio of 91:9 had the highest calcium concentration (1,113.0 ppm), whereas the CF formulation with Mocaf only had the lowest (662.5 ppm) as shown in Figure 3. The content of fish meal in analog rice CF can increase the calcium content. The recommended calcium requirement for osteoporosis control is between 700 and 1,200 mg per day. The consumption of more than 2,000 mg per day can cause hypercalcemia (Cano et al., 2018; Pu et al., 2016).

There were statistical differences in protein content among the CF formulations (as shown in Table 2). The CF formulation with Mocaf:SF ratio of 88:12 had the highest protein content (11.64%), whereas CF with Mocaf only had the lowest (7.21%). The difference in protein content is influenced by the amount of SF content in CF. An adult's protein requirement is >25 g protein per serving, where male protein requirements are more significant than those of women (Mishra et al., 2017). In elderly people, cell function efficiency will begin to decrease and nutritional needs will increase (Baugreet et al., 2017). Protein stimulates insulin secretion so that glucose in the blood can be well controlled. Therefore, food with high protein content has a lower glycemic index value than food with low protein content (Aller et al., 2014).

The fat content in analog rice from Mocaf and cornstarch fortified calcium from snakehead fish varied (Table 2). The lowest fat content was in sample 3 (94:6) with 0.54% and the highest in sample 15 (88:12) with 1.28%. This fat content is lower than commercial analog rice containing approximately 2.18% of fat. Consumption of foods containing fat needs to be carefully considered because the total limit of fat consumption should not exceed 30% of the total energy needs (Brouwer et al., 2010). Indeed, excess fat consumption can lead the body to various diseases, including obesity (Brouwer et al., 2010; Muka et al., 2017). Obesity may also indirectly increase the risk of decreased bone strength and osteoporosis (Fujita, 2018). For the same extrusion temperature, the fat content in fish (in its CF) makes the fat content in rice proportional to fish meal content.

From the results of our analyses, it was found that the highest dietary fiber content (4.41%) was found in sample 12 (97:3), whereas the lowest crude fiber content (2.43%) was found in sample 9 (91:9) (as shown in Table 2). These results show that the analog rice studied contains less dietary fiber than commercial analog rice containing 7.05% dietary fiber. Based on the composition, the higher amount of fish meal used, the lower the fiber content. For elderly people, fiber consumption patterns may affect psychological activity, metabolism, and microbiome function (Wei et al., 2018).

### 3.3 Extrusion temperature

Based on the gelatinization temperature, the extrusion process was carried out at 50, 60, 70, 80, and 90°C, and subsequently, the effect on analog rice's nutrient content was investigated through proximate analyses. Table 4 shows the effect of the extrusion temperature on the analog rice nutritional content.

**TABLE 4:** Influence of extrusion temperature on analog rice nutritional content

Parameter	Extrusion Temperature (°C)		
	50	70	90
Protein (% wt)	8.82 ± 0.02	9.61 ± 0.02	9.03 ± 0.02
Carbohydrate (% wt)	69.51 ± 0.7	71.04 ± 0.4	73.43 ± 0.8
Fat (% wt)	0.73 ± 0.003	0.89 ± 0.002	0.92 ± 0.003
Moisture (% wt)	16.50 ± 0.05	14.34 ± 0.02	11.93 ± 0.04
Ash (% wt)	0.71 ± 0.003	0.99 ± 0.006	0.90 ± 0.001
Calcium (ppm)	908.00 ± 6.7	1,113.00 ± 10.4	892.50 ± 7.5
Dietary Fiber (% wt)	3.66 ± 0.03	3.13 ± 0.05	3.80 ± 0.01

**FIGURE 4:**
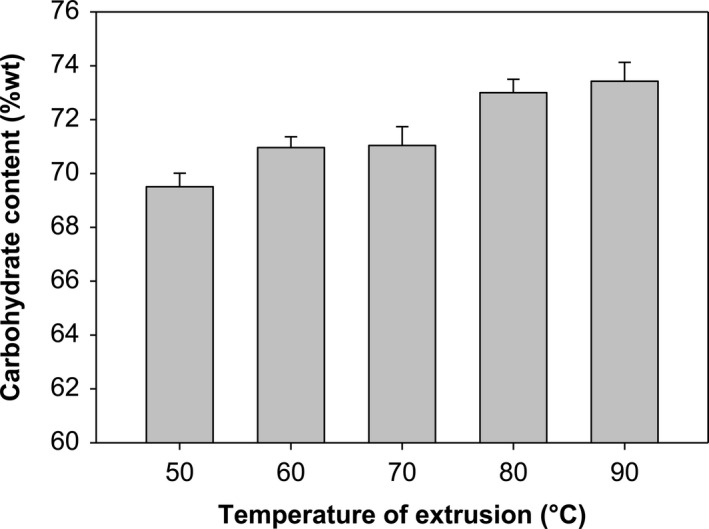
Influence of extrusion temperature on carbohydrate content (with CF ratio 91:9)

Analog rice carbohydrate analysis results from temperature influence are represented in Figure 4. Increasing the extrusion temperature affects the carbohydrate content of analog rice; the higher the extrusion temperature will cause an increase in the carbohydrate content of analog rice. High temperatures will easily separate amylose and amylopectin molecule chains increasing the number of amylose molecules and amylopectin. The number of amylose and amylopectin molecules in analog rice indicates that carbohydrate levels are also high (Wulan et al., 2007).

**FIGURE 5:**
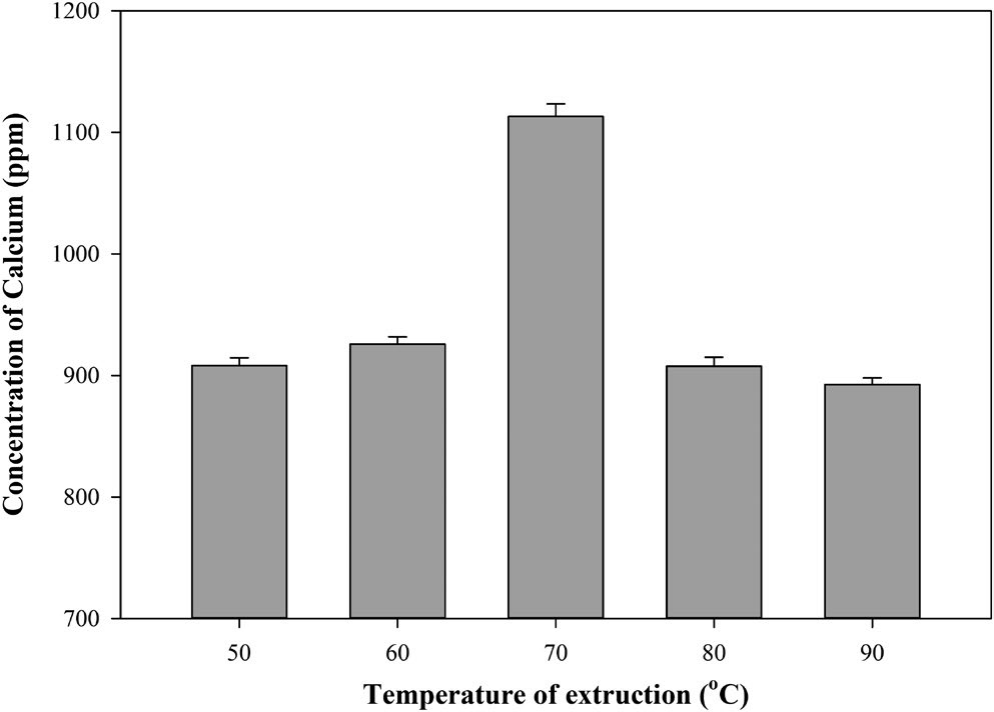
Influence of extrusion temperature on calcium concentration (with CF ratio 91:9)

The results of calcium analysis on the effect of extrusion temperature on analog rice are represented in Figure 5. Increasing the extrusion temperature causes increased levels of calcium. The gelatinization process absorbs calcium minerals from SF and nourishes analog rice (Da Silva et al., 2017). However, at a temperature of 90°C, there was a decrease in calcium levels from 1,113.00 ppm to 892.50 ppm. Declined levels of calcium at high temperatures correlate with the gelatinization temperature of CF. The gelatinization temperature of CF is 80.7°C. Higher extrusion temperatures cause higher amylose levels, denser amylose structures, and reduced water absorption capability. Higher extrusion temperatures resulted in the difficulty of water penetrating rice and lower absorption of calcium in analog rice. Besides, the high‐extrusion temperature may lead to amylose dissolution and damage to the amylose crystals. If the heating continues, amylose will be leached, and subsequently, when leaching occurs, the calcium that has been absorbed will be transported out of the tissue (Wariyah et al., 2008).

At different extrusion temperatures, results showed that the protein levels of analog rice ranged from 8.82% to 9.61%. The highest protein content was obtained at 70°C and the lowest at 50°C. Increasing the extrusion temperature causes will increase protein level, but at 90°C, the level decreases. High temperatures lead to reduced protein levels in analog rice due to protein denaturation. The mechanical process of extrusion and heat addition causes the breaking of amino acid bonds, except for the primary bond, and breaks down hydrogen bonds and nonpolar hydrophobic interactions in analog rice. High temperatures increase the kinetic energy and cause protein molecules to move very quickly so that the amino acid bonds in the protein molecule are split. Breaking amino acid bonds in proteins causes protein denaturation so that the protein content in analog rice decreases (Bender, 2012).

The analysis result of analog rice fat due to extrusion temperature influence ranged from 0.73% to 0.92%. Increased extrusion temperature increases the fat content of analog rice. For the same cornstarch composition in CF, higher extrusion temperatures can maintain better fat content so that the fat content is higher than the low extrusion temperature. This phenomenon is due to the decrease in analog rice moisture content during the extraction process because if any one of the proximate components of food decreases, then other proximate components will increase to achieve balance (Michalczuk et al., 2016).

The result of dietary fiber analysis on analog rice temperature variables ranged from 3.13% to 3.80%. Dietary fiber content decreased with an increase in extrusion temperature from 50 to 70°C. The decrease in dietary fiber is due to the decay of cell walls of analog rice during extraction (Liam et al., 2014). However, there was an increase in the dietary fiber content at 90°C. Increased levels of crude fiber are thought to occur due to decreasing moisture content in the analog rice. During the extraction process, water in the analog rice will evaporate, but the composition of other compounds, such as carbohydrates, increases. As carbohydrate levels rise, the ingredient's coarse fiber content will increase (Li et al., 2019).

The moisture content analysis results on analog rice's at different extrusion temperatures ranged from 11.93% to 16.50%. The variation of temperature shows that the higher the extrusion temperature, the lower the analog rice's moisture content. The extrusion temperature of 50°C is low, the moisture content in CF will be high, and ultimately in the analog rice. At 90°C, some of the water in the CF will evaporate during extraction such that when dried, the moisture content will be low. The discharge of water in the extraction process results in decreased moisture content (Brahma et al., 2017).

### 3.4 Physical analysis of analog rice

#### 3.4.1 Bulk density

Based on the average density analysis on analog rice, the value of bulk density was 0.51 g/ml, lower than Mocaf‐based analog rice density of 0.70 g/ml. Based on these results, rice from Mocaf, cornstarch, and snakehead fish composites has a smaller weight than Mocaf‐based analog rice at the same volume. The increase in analog rice porosity is influenced by analog rice's nutrient content and the manufacturing process, including drying. The drying process causes the analog rice to lose water to become more porous (Sumardiono et al., 2014). During the cooking process, there is development or expansion. The development or expansion of rice during cooking increases the rice volume but decreases the mass. Therefore, the higher the rice expansion rate, the lower the bulk density (Sumardiono et al., 2018). However, American government specifications in the military and defense fields set the standard for rice cages density ranging from 0.40 to 0.42 g/ml. The low bulk density of rice will produce flabby products such as rice porridge at the time of reconstitution (Virdi et al., 2019). Based on these data, it can be concluded that the analog rice from Mocaf, cornstarch, and snakehead fish composites produced from this study falls within the criteria according to Yu et al. (2017).

#### 3.4.2 Cooking time

The analysis of cooking time on analog rice from Mocaf, cornstarch, and snakehead fish composite takes 25–30 min, longer than Mocaf‐based analog rice, which is only 15 min. The analog rice from Mocaf, cornstarch, and snakehead fish composites has high protein content, affecting analog rice cooking time. Higher protein value will require more heat energy to obtain gelatinization because heat energy is used for protein denaturation (Pudjihastuti et al., 2018). It can be concluded the more significant the protein content, the longer the optimum time of cooking.

### 3.5 Acceptance response of analog rice consumers

**FIGURE 6:**
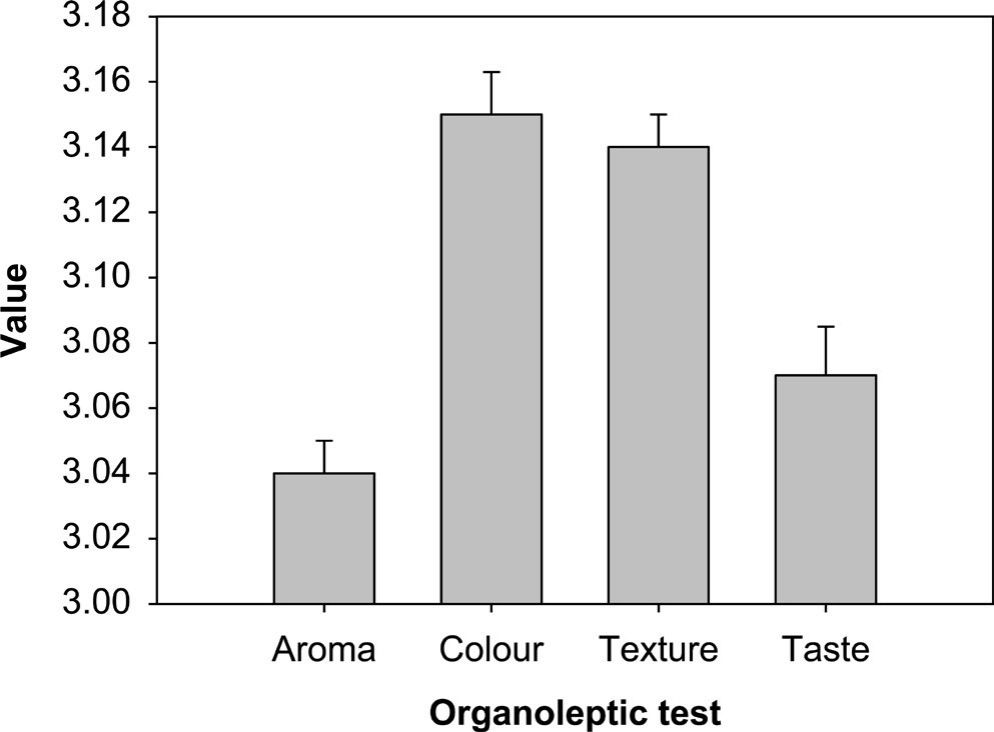
Organoleptic test result of analog rice

The organoleptic test of analog rice was analyzed using the consumer's acceptance test method using the parameters of texture, aroma, taste, and color. This test had got 187 responses from 42 respondents conducted randomly. The analog organoleptic test showed consumers’ acceptability to analog rice made with the best formulation previously selected. The best analog rice formulation (sample 9, 91:9) was determined based on the best proximate analysis of various compositions. Respondents were assessed for their analog rice preference using a 1–5 scale ranging from “very much did not like” to “very much like.” The results of organoleptic tests on analog rice are presented in Table 5 and Figure 6.

**TABLE 5:** Organoleptic test results of analog rice^a^

Parameter	Number of responses for each score					Average
	1 (very much disliked)	2 (did not like)	3 (neutral)	4 (liked)	5 (very much liked)	
Aroma	13	34	78	51	7	3.04
Taste	16	37	63	54	13	3.07
Color	6	34	80	54	9	3.15
Texture	11	36	66	60	10	3.14

^a^ Organoleptic test using analog rice sample no.9 (91:9).

Based on Table 5, for each parameter, most of the respondents gave ratings on the value 3, which suggests that respondents can receive analog rice as a substitute for ordinary rice. Analog rice that has been flavored has an odor of fish as it is produced from SF, a relatively bland taste, was brownish in color, and relatively coarser in texture than ordinary rice: However, it was still acceptable to the respondents.

## 4 CONCLUSIONS

In conclusion, based on the proximate analysis on various compositions of CF, sample 9 (with a ratio of Mocaf to CF of 91:9 and extrusion temperature of 70°C) is the analog rice with the best formulation. The highest calcium and carbohydrate levels in this sample of analog rice could make it a staple food.

## CONFLICT OF INTEREST

The authors declare that they do not have any conflict of interest.

## AUTHOR CONTRIBUTIONS

**Siswo Sumardiono:** Conceptualization (lead); Formal analysis (equal); Funding acquisition (lead); Investigation (equal); Methodology (lead); Project administration (lead); Resources (equal); Software (supporting); Supervision (lead); Validation (equal); Visualization (equal); Writing‐original draft (lead); Writing‐review & editing (equal). **Budiyono:** Conceptualization (equal); Formal analysis (equal); Investigation (equal); Methodology (equal). **Heny Kusumayanti:** Data curation (equal); Funding acquisition (supporting); Investigation (supporting); Project administration (equal); Resources (equal). **Novian Indra Agung Prakoso** **:** Data curation (equal); Investigation (equal); Validation (equal); Writing‐original draft (equal). **Fawzia Puti Paundrianagari:** Data curation (equal); Investigation (equal); Validation (equal); Writing‐original draft (equal). **Heri Cahyono:** Formal analysis (equal); Software (lead); Writing‐review & editing (equal).

## ETHICAL APPROVAL

Ethical Review: This study was approved by the Institutional Review Board of Universitas Diponegoro.

Informed Consent: Written informed consent was obtained from all study participants.
